# DEM analysis of boundary effects in simple shear tests

**DOI:** 10.1038/s41598-026-37235-1

**Published:** 2026-03-06

**Authors:** Jikai Guo, Mengchen Sun, Michelle L. Bernhardt-Barry, Giovanna Biscontin

**Affiliations:** 1 SINOPEC Research Institute of Petroleum Engineering, Beijing, China; 2 University of Emergency Management, Langfang, China; 3 Department of Civil Engineering, University of Arkansas, Fayetteville, AR USA; 4 U.S. National Science Foundation, Alexandria, VA USA

**Keywords:** Discrete element method (DEM), Granular response, Simple shear testing, Boundary effects, Engineering, Mathematics and computing

## Abstract

In simple shear testing, the specimen boundaries play a pivotal role in the transmission of shear forces. Waffle-style porous stones, plates with ribs or similar types of projections are used in experiments to reduce slippage at the boundary and transmit shear throughout the specimen. Conventionally, Discrete Element Method (DEM) simulations often model the top and bottom caps as flat boundaries with artificially enlarged friction coefficients, or use geometrical configurations that are computationally efficient. This research compares flat high-friction boundaries with ribbed and more novel-designed boundaries incorporating large pyramid, and small pyramid projections, aiming to improve shear transmission capability while ensuring computational efficiency. DEM simulations were conducted on specimens of steel bearings, with experimental validation using identical setups and particle properties. Specimens featuring different boundaries showed different void ratios in the layers close to the boundaries, with boundary effects diminishing towards the central zones. DEM simulations with projection boundaries demonstrated good agreement with experiments in terms of the macroscopic response, validating the effectiveness of the projection boundaries. The conventional flat boundaries exhibited limited shear transmission capability, resulting in insufficient development of shear stress and inadequate particle engagement. Conversely, projections on boundaries significantly improved shear stress transmission, ensuring the simple shear condition throughout the entire specimens. Projection boundaries introduced manageable increases in computational cost despite increased mesh complexity. This study highlights the importance of boundary design and recommends the adoption of projection-based boundaries in both experimental and numerical simple shear tests to ensure effective shear transmission and reliable results.

## Introduction

The ability to replicate the in-situ rotation of principal stresses makes the simple shear test a useful method for determining soil shear strength. The NGI-type device with a cylindrical specimen, first developed by the Swedish Geotechnical Institute in 1936 and later modified at the Norwegian Geotechnical Institute^1,2^, has become more prevalent due to its commercial availability and ease of sample preparation. In an NGI-type experiment, the specimen is enclosed by a wire-reinforced membrane or a stack of rings to provide lateral confinement. The shear force is transmitted by top and bottom flat caps with sufficient roughness to transfer shear stress, as suggested by ASTM-D6528^3^, with porous stones recessed into them for drainage. However, when testing specimens such as sands, steel bearings, or glass beads, slippage along the boundaries can occur, leading to insufficient shear stress transmission, an inability to measure shear strength accurately, and non-uniform stress and strain distributions throughout the specimen^4–7^.

For element-level tests like simple shear tests, specimen boundaries play a crucial role and can significantly impact macroscopic and microscopic results. Numerous studies have investigated boundary effects through discrete element method (DEM) simulations, including works by Chan and Ng^8^, Cundall^9^, Pöschel and Schwager^10^, Marketos and Bolton^11^, and Zhang and Evans^12^. While these DEM studies have been effective in studying idealized specimens and fundamental granular mechanics, actual experimental boundary conditions can be more complex and may not be adequately described by rigid flat boundaries or periodic boundaries.

Attempts have been made to validate simple shear tests using DEM with experimental calibration. Wijewickreme et al.^13^ and Dabeet et al.^7^ used flat boundaries with a significant friction coefficient of 10 and selected an interparticle friction coefficient equal to that of glass beads based on parametric studies. Zhang et al.^14^ also simulated glass beads in simple shear tests, but the specifics of the top and bottom boundaries were not mentioned. Asadzadeh and Soroush^15^ used a saw-tooth configuration for shear transmission on the top and bottom boundaries which matched the experimental setup used. Bernhardt et al.^16^ and Bernhardt-Barry^17^ employed fixed particles on the top and bottom boundaries, with particles adjacent to the caps virtually glued by setting their velocity equal to that of the caps. This method, also shown effective in transferring shear stress by Stroud^18^ and Shen^19^, may encounter durability issues in experiments, as particles glued at boundaries may dislodge during the tests. This method was also very computationally expensive when stress-control algorithms were used. Therefore, developing a numerically efficient boundary treatment able to ensure sufficient shear transmission in both closely matching numerical and experimental setups is essential.

This study aims to evaluate different types of rigid boundaries, including flat boundaries and boundaries with projections to assess the efficiency of shear transmission. Microscopic behavior will also be evaluated to better understand shear transmission mechanisms by analyzing particle displacements and rotations. This study closely compares DEM models and experimental results using identical particle properties and testing setups. The simulation results can provide valuable insights for refining future simple shear tests on granular materials.

## Description of DEM models

**Fig. 1:**
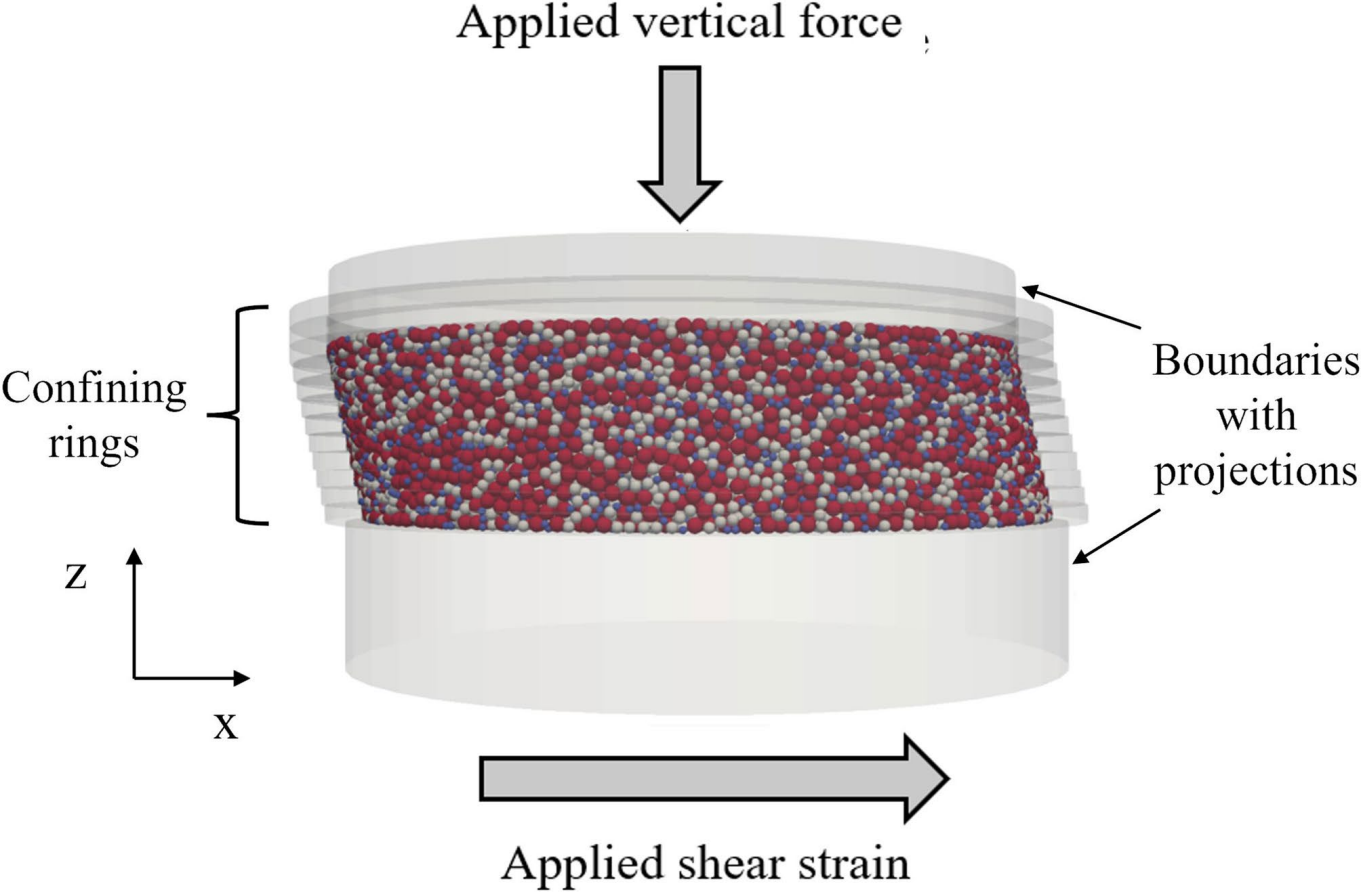
Schematic of specimen describing load condition and boundaries.

This study was conducted using the open-source software LIGGGHTS, running on High-Performance Computing (HPC) facilities, with the basic parallel algorithms described by Kloss et al.^20^. The simplified Hertz model was applied to calculate movements at particle contacts, with all the input parameters listed in Table 1. The DEM specimen was generated and prepared to match the dimensions of experimental tests using the multi-directional simple shear apparatus (MDSS) developed by Rutherford and Biscontin^21^. The schematic of the specimen setup is shown in Fig. 1, featuring a cylindrical shape with a diameter of 101.6 mm and a height of approximately 25 mm, enclosed by two caps specially designed with 3D projections to ensure shear transmission, along with twelve confining rings. In the DEM model, the top and bottom caps, as well as the confining rings, were set as fixed boundaries so that particles in contact with these boundaries could directly transfer forces. The specimen comprised 60,000 spherical particles of three different diameters: 1 mm, 1.5 mm, and 2 mm, with 20,000 particles in each size group.

**Table 1:** Simulation parameters.

Parameter	Samples using flat boundaries	Sample using boundaries with projections
Density, $\:\rho\:$ (kg/m^3^)	7800	7800
Young’s modulus, $\:E$ (GPa)	200	200
Poisson’s ratio, $\:{\upnu\:}$	0.3	0.3
Coefficient of friction, interparticle, $\:{\mu\:}_{\mathrm{p}}$	0.096	0.096
Coefficient of friction, particle-ring interface, $\:{\mu\:}_{\mathrm{r}}$	0.36	0.36
Coefficient of friction, particle-cap interface, $\:{\mu\:}_{\mathrm{c}}$	10	0.44
Timestep, $\:t$ (s)	1 × 10^− 7^	1 × 10^− 7^

During sample preparation, a cloud of non-contacting particles was generated above the bottom cap. Gravity was then applied to the entire system following procedures similar to the air-pluviation methods used in laboratory tests^22,23^. A sufficient number of time steps was applied to ensure all the particles were completely settled as measured by the out-of-balance force. Different specimen densities were achieved by varying the interparticle friction coefficient, $\:{\mu\:}_{\mathrm{p}}$, during the settling process. For a dense specimen, $\:{\mu\:}_{\mathrm{p}}$, was set to zero during settling, with additional time steps for further settling, and then switched back to 0.096 matching the experimental coefficient before vertical stress application. A constant $\:{\mu\:}_{\mathrm{p}}$ of 0.096 was used for loose specimens during settling and consolidation. Consolidation was achieved by placing the top cap onto the specimen and slowly increasing the vertical stress to 100 kPa, while preventing any horizontal deformations at the lateral boundary. A specimen height of approximately 25 mm was achieved for each specimen at the end of consolidation.

During the shearing stage, the applied vertical stress was kept constant at 100 kPa and the top boundary was allowed to move only vertically. The simple shear condition during shearing was achieved by generating a linear strain profile at side boundaries. The top cap was fixed, and the bottom cap moved at a constant shear rate, while the top and the bottom rings were attached to their respective caps, and the other rings moved with proportional velocities in between. During shearing, ring forces were monitored to ensure they remained close to zero, consistent with the physical tests. A velocity equivalent to 1 mm/s was maintained for the bottom cap to achieve a relatively fast simulation while ensuring the monotonic shearing of the specimen under a quasi-static condition^24^.

**Fig. 2:**
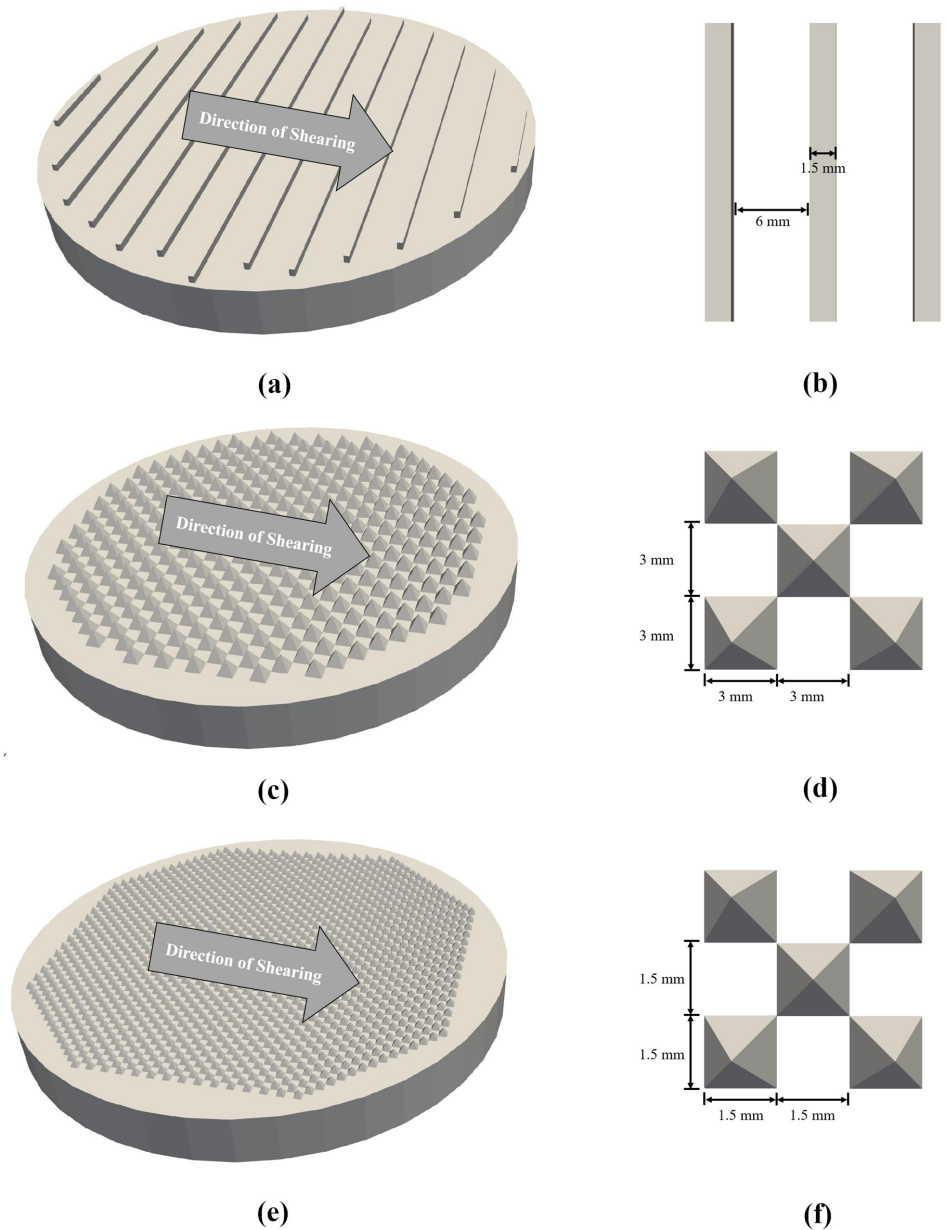
Projections on bottom boundaries; (**a**) isometric view of a ribbed cap; (**b**) top view of a group of ribs (each rib projection is 1.5 mm in height); (**c**) isometric view of a large pyramid cap; (**d**) top view of a group of large pyramids (each pyramid projection is 3 mm in height); (**e**) isometric view of a small pyramid cap; (**f**) top view of a group of small pyramids (each pyramid projection is 1.5 mm in height).

The conventional flat porous stones on the caps had limited shear force transfer ability, especially for cohesionless specimens. Gluing particles to the caps improved shear transmission in both simple shear experiments and DEM simulations^16–19^. However, measuring the total volume of spheres combined with epoxy posed challenges in void ratio calculation, and some glued spheres may be lost during or after repeated tests, altering boundary conditions and shear transmission. Most importantly, fixed boundary particles in the DEM simulations significantly increased the computational cost when stress control algorithms were used. In this study, 3D-printed plates with projections replaced the porous stones on caps, fabricated using PLA filament through the Fused Deposition Modeling (FDM) process. A schematic of the bottom caps with different projections and their dimensions is shown in Fig. 2. Three types of projections, including ribbed patterns and two types of pyramid projections, were used in the DEM and experimental tests for validation. Furthermore, DEM simulations using flat caps with artificially high boundary friction coefficients ($\:{\mu\:}_{\mathrm{c}}$ = 10) were also conducted for comparison.

In LIGGGHTS, the caps and confining rings were input as walls with triangular meshes from ASCII STL files, with a larger number of meshes caused by projections. High-performance computing with Skylake nodes at the University of Cambridge was used in this study, but it should be noted that computational time can vary across clusters. For 60,000-particle specimens, one computational node and 32 CPUs were optimal for running simple shear tests in LIGGGHTS. Table 2 details the computational time to reach 15% shear strain. Despite the number of particles and contacts, the speed of simulation is also highly dependent on timestep, $\:t$ and shear strain rate, $\:\dot{{\varepsilon}_{q}}$, which are often selected by maintaining the quasi-static condition and ensuring acceptable particle overlaps^24,25^. For all the simulations, $\:t$ and $\:\dot{{\varepsilon}_{q}}$ were set to 1 × 10^− 7^ s and 1 mm/s, respectively. Overall, running such a stiff specimen in DEM incurred significant computational cost, with simulations using flat caps requiring slightly less time than those with projections. Ribbed caps increased computational time slightly, while large and small pyramid caps increased it by 2% and 6%, respectively. Despite the increased mesh count with projections, the added computational cost was manageable.

**Table 2:** Computational time to run 15% shear strain using 32 CPUs.

Boundary type	Sample name	Number of triangular meshes	Time to run 15% shear strain (hours)
Flat	FC-D	328	140.6
	FC-L	328	141.4
Ribbed	RC-D	744	141.2
	RC-L	744	141.9
Large pyramid	PC1-D	2,342	145.8
	PC1-L	2,342	143.6
Small pyramid	PC2-D	19,600	150.20
	PC2-L	19,600	149.73

## Boundary effects on specimens during sample Preparation

**Fig. 3:**
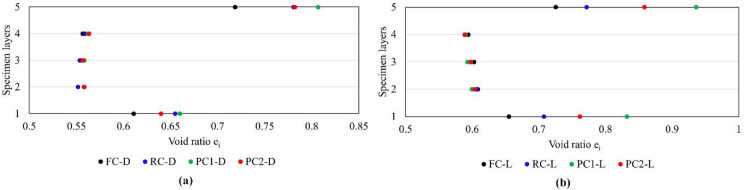
Void ratios of different layers in DEM simulations for caps with different boundaries; (**a**) dense specimens; (**b**) loose specimens.

Table 3 lists the DEM simulations and the initial void ratio ($\:{e}_{0}$) of each DEM specimen under 100 kPa vertical stress before shearing, with $\:{e}_{0}$ varying depending on different caps. The calculation of $\:{e}_{0}$ considered the volume occupied by projections, and the difference in magnitudes is only due to the features of the boundaries. Each specimen was virtually subdivided into five horizontal slices with equal thickness, each about 5 mm to track changes in void ratio across the specimen separately. Figure 3 displays the void ratio of each layer ($\:{e}_{\mathrm{i}}$) in dense and loose specimens with different caps. For each type of boundary, a larger $\:{e}_{\mathrm{i}}$ could be observed for the layers adjacent to the boundary, which matched the observation by Chan and Ng^8^. One of the key requirements for good sample preparation as discussed by Stroud^18^ is the uniform void ratio distribution. The values of $\:{e}_{\mathrm{i}}$ were consistent in the middle three layers, as shown in Fig. 3a,b, agreeing well with the fact that the specimens were prepared following the same procedure. The variation in the overall initial void ratio ($\:{e}_{0}$) was solely due to different boundaries, where caps with projections created larger void ratios at boundaries compared to flat caps. For dense specimens, similar $\:{e}_{\mathrm{i}}$ values were found at boundaries with projections, whereas for loose specimens, the difference was more pronounced. In particle assemblies with low coefficient of uniformity, i.e., similar particle sizes, any hard boundary creates a higher void ratio because particles are prevented from fitting more snugly into the spaces. The large pyramid projections resulted in the most significant voids at the top and the bottom boundaries, indicating less penetration into the specimen due to pyramid dimensions and shape. These findings suggest that similar patterns are likely in physical specimens prepared following the same procedures, where only a global void ratio can be calculated.

**Table 3:** Specification of DEM simulations.

Sample description	Sample name	Initial void ratio, $\:{e}_{0}$
Flat caps, dense	FC-D	0.598
Flat caps, loose	FC-L	0.636
Ribbed caps, dense	RC-D	0.613
Ribbed caps, loose	RC-L	0.649
Large pyramid caps, dense	PC1-D	0.621
Large pyramid caps, loose	PC1-L	0.655
Small pyramid caps, dense	PC2-D	0.614
Small pyramid caps, loose	PC2-L	0.646

**Fig. 4:**
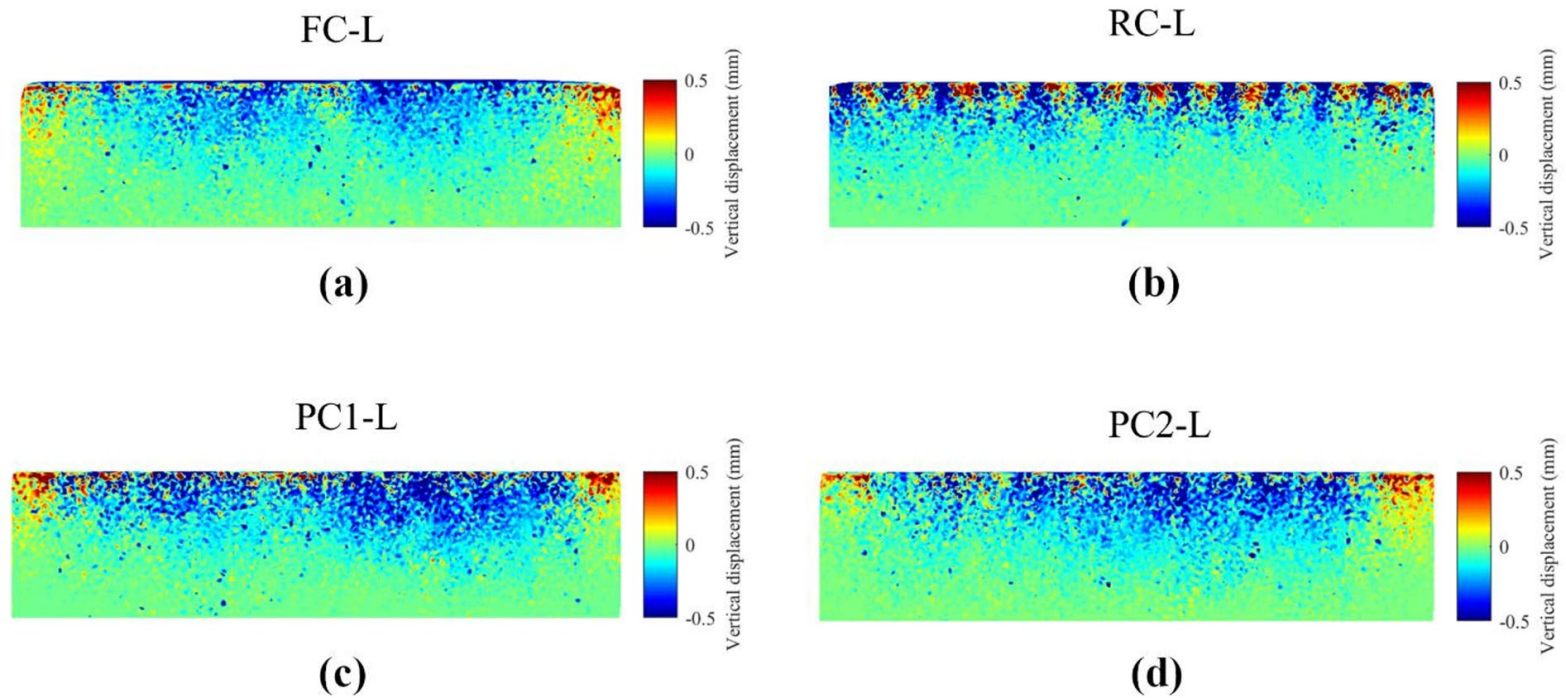
Vertical displacement in loose specimens during the consolidation stage from 0 kPa to 100 kPa.

The shape of the boundaries also influenced particle movement patterns during the consolidation stage, which can be examined from a microscopic perspective. Under the application of monotonic vertical stress from the top cap, and with the confining rings providing lateral restraint to maintain K_0_ conditions, the particles tended to move downward and form a denser packing. Figure 4 illustrates the vertical particle displacement at the end of the consolidation stage for loose specimens, offering a microscopic view of the specimen response as the normal stress increased to the target value for simple shear testing, which is an aspect not explored in previous studies. Ribbed caps exhibited the same pattern with more pronounced movements, but at a smaller scale localized around the ribbed projections. The ribs penetrated into the specimen and particles were displaced upwards between ribbed projections. Another requirement for a well-prepared sample as suggested by Stroud^18^ is that the full frictional contacts between the top surface of sand and the face of the top cap should be developed, which was facilitated by the sliding and penetration of projections through the particles at the top layers by the projection caps. In contrast, samples with flat boundaries were unable to generate enough friction due to the lack of sliding between the boundaries and particles during consolidation.

## Experimental validation of DEM tests

Caps with the same designs were used in physical experiments for direct comparison with the DEM simulations. Specimens were prepared in dense and loose states, with numerous tests conducted to create a comprehensive database for validating DEM results. The variations of initial void ratios ($\:{e}_{0}$) with different types of boundaries are presented in Table 4. Specimens were prepared using air-pluviation of particles into a sieve with meshes placed in contact with the bottom cap and within the confining rings, similar to the methods employed by Stroud^18^, Vaid and Negussey^26^, Wijewickreme et al.^27^ and Bernhardt et al.^16^. To prepare a loose specimen, the sieve was slowly raised to minimize disturbance, followed by gentle placement of the top cap. For dense specimens, after setting the top cap, densification was achieved by tapping and vibrating. The specimen was then placed into the simple shear device for testing. The measured specimen heights under the vertical stress of 100 kPa at the end of consolidation were used to calculate the initial void ratios, $\:{e}_{0}$.

**Table 4:** Void ratios of experimental tests.

Boundary type	Sample category	Range of initial void ratios, $\:{e}_{0}$
Ribbed	Dense	0.590–0.600 (6 tests)
	Loose	0.630–0.640 (6 tests)
Large pyramid	Dense	0.600-0.616 (4 tests)
	Loose	0.651–0.663 (6 tests)
Small pyramid	Dense	0.596–0.620 (9 tests)
	Loose	0.650–0.665 (6 tests)

Specimens with large pyramid and small pyramid caps showed similar values of $\:{e}_{0}$ across tests, averaging about 0.61 for dense and 0.66 for loose specimens. For the specimens using ribbed caps, the average $\:{e}_{0}$ of dense and loose specimens were around 0.595 and 0.635, respectively. The difference in $\:{e}_{0}$ for specimens with different caps was more pronounced in the experiments compared to the DEM simulations likely due to the occurrence of experimental error. In the experiments, the top cap may tilt or rock slightly when placed on the specimen, leading to errors in measuring $\:{e}_{0}$. This impact was more significant in the specimens with ribbed caps because more particle penetrations were required to position the cap onto the specimen, as visualized in Fig. 4b.

**Fig. 5:**
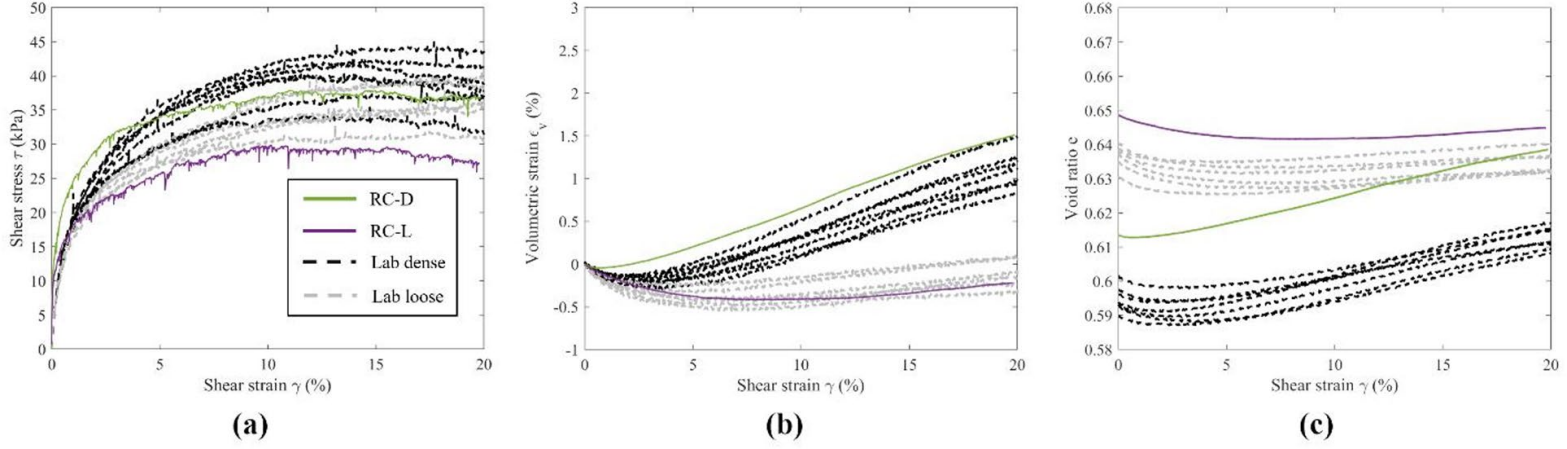
Macroscopic response of specimens with ribbed caps, DEM vs. LAB.

**Fig. 6:**
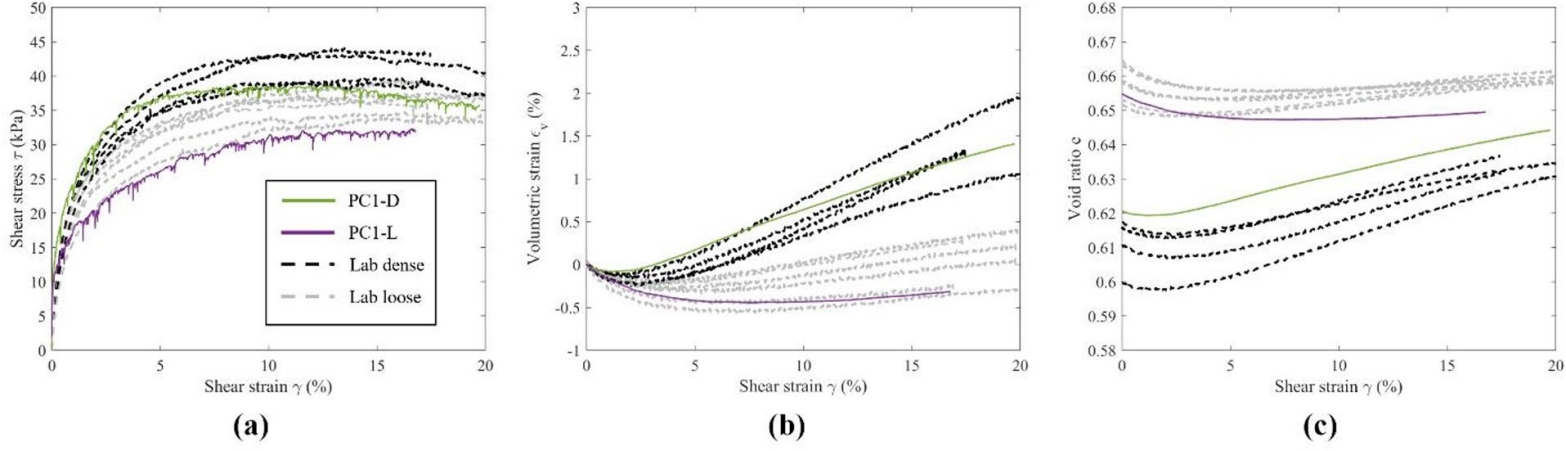
Macroscopic response of specimens with large pyramids caps, DEM vs. LAB.

**Fig. 7:**
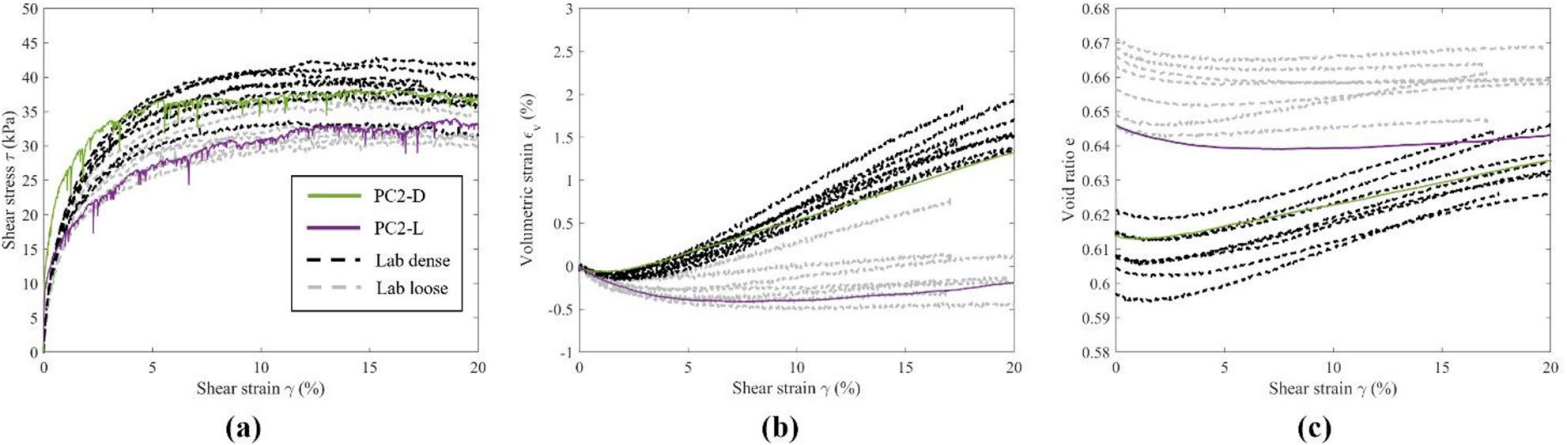
Macroscopic response of specimens with small pyramids caps, DEM vs. LAB.

Figures 5, 6 and 7 depict the macroscopic stress–strain and volumetric responses of dense and loose DEM specimens using ribbed, large, and small pyramid caps, validated by extensive experimental tests with the same type of caps. Direct comparison of simulation results to experiments validates macroscopic responses, aiding analysis of particle-level information from the DEM simulations. Overall, the DEM results captured the behavior of the granular assemblies and matched the macroscopic responses observed in the experiments. Shear stress, $\:\tau\:$, and volumetric strain, $\:{\varepsilon}_{v}$, responses aligned closely between the DEM simulations and the experiments, particularly at large $\:\gamma\:$. Differences in initial stiffness were noted, with the DEM specimens showing rapid development of $\:\tau\:$ and lower initial contractive response, converging with those of the experiments with further development of $\:\gamma\:$ (5% for dense and 2% for loose).

The differences in $\:e$ between the DEM simulations and the experiments were notable, as shown in Figs. 5c, 6c and 7c. In general, a smaller range of $\:e$ between dense and loose specimens could be observed for the DEM specimens. At large $\:\gamma\:$, dense and loose DEM specimens approached similar $\:e$, as expected for granular materials when approaching the critical state. For specimens with ribbed caps, as shown in Fig. 5c, DEM dense and loose specimens exhibited larger values of $\:e$ than the experiments. For DEM specimens with large and small pyramid caps as shown in Figs. 6c and 7c, the dense and loose $\:e$ were in between those of the experiments. The discrepancy between experimental and DEM $\:e$ could be due to experimental errors as mentioned earlier, as well as vertical compliance. For instance, the magnitude of the experimental void ratio was sensitive to small errors in the measurement of specimen height, which was calculated from the vertical displacement transducer. A 0.01 difference in $\:e$ was reflected by around 0.15 mm in height measured by the transducer, which was approximately 1/10 the average diameter of a single steel sphere. Nevertheless, the physical tests in the range of the void ratios calculated from boundary measurements still responded across the behavior expected from dense to loose states, ensuring comparability to the DEM results. Based on the macroscopic responses, the DEM models effectively replicated the range of responses in the physical simple shear tests, indicating reliability for further analysis.

## Comparison of shear transmission capabilities with different boundaries

In conventional simple shear experiments, porous stones are commonly used on top and bottom boundaries, however, for specimens consisting of steel spheres with smooth particle surfaces, flat boundaries are ineffective in transmitting shear stress efficiently to the specimen. In DEM simulations of simple shear tests, one approach is to artificially increase the friction coefficient on the top and bottom caps, as proposed by Wijewickreme et al.^13^. However, employing a high boundary friction coefficient deviates from the actual contact physics between the boundary and spheres, making direct validation through experiments challenging. Consequently, the performance of the flat boundaries with significant friction can only be assessed numerically.

**Fig. 8:**
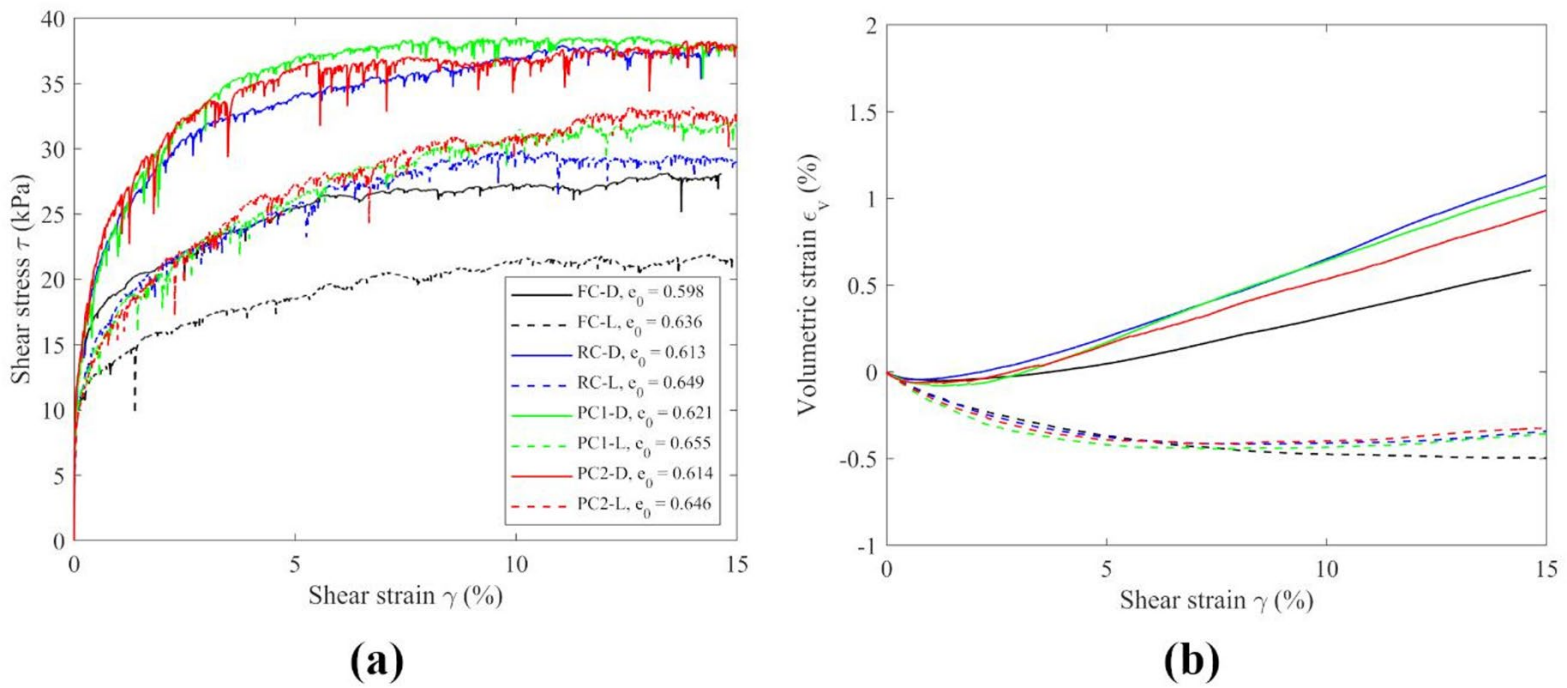
DEM simulations of simple shear tests with four types of boundaries. Comparison of macroscopic response.

Dense and loose DEM specimens with flat boundaries were prepared similarly to those with projection caps. However, during the shearing stage, the friction coefficient, $\:{\mu\:}_{\mathrm{c}}$, between particles and boundaries was set to 10, as indicated in Table 1, to simulate very rough top and bottom caps. Figure 8 presents the macroscopic responses of specimens with flat caps alongside results from numerical tests using projection caps. As discussed earlier in Fig. 3, different boundaries resulted in varied $\:e$, although the $\:{e}_{\mathrm{i}}$ within the middle 3/5 of specimens were found to be similar. In Fig. 8a,b, comparable magnitudes of $\:\tau\:$ and $\:{\varepsilon}_{\mathrm{v}}$ can be observed for the specimens with projection caps. However, specimens with flat caps (FC-D and FC-L) exhibited lower stiffness and $\:\tau\:$ throughout the shearing stage. In terms of volumetric strain, $\:{\varepsilon}_{v}$, similar values and trends were observed for all the specimens where FC-D showed slightly less dilation compared to other dense specimens, while FC-L displayed greater contraction towards the end of shearing compared to other loose specimens approaching a steady state. Hence, the flat boundaries used in simple shear tests demonstrated lower shear transmission capabilities than projection caps.

**Fig. 9:**
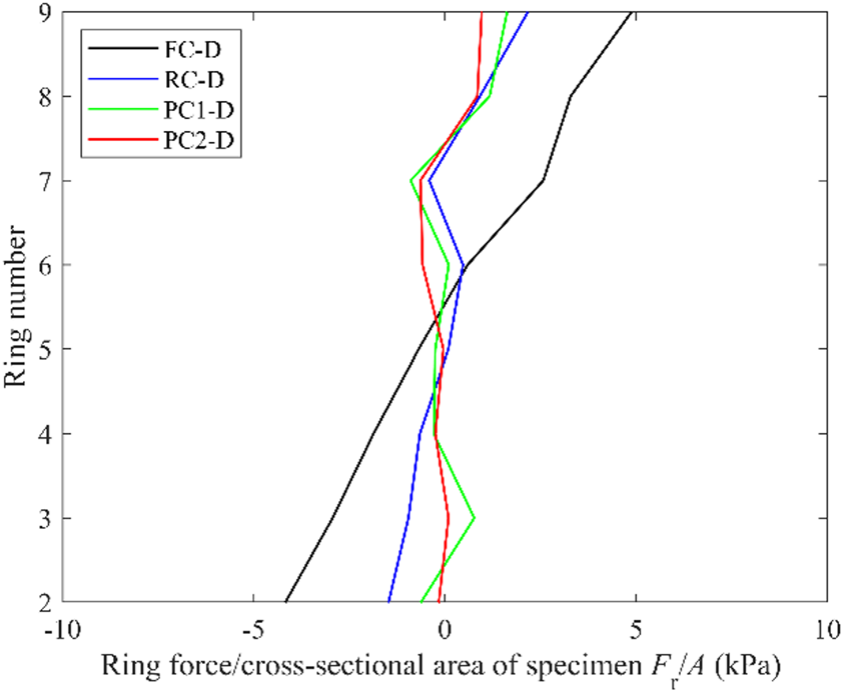
Ring forces normalized by the specimen cross-sectional area for all dense DEM simulations at 15% shear strain.

In physical simple shear tests, the shear force is ideally fully transmitted by the caps, resulting in the net force of zero on confining rings or the membrane. The top and bottom rings (ring 1 and ring 10) in the DEM simulations are attached to the top and bottom caps during shearing, while all the other rings (ring 2 to ring 9) move with intermediate velocities following a linear profile. Figure 9 presents the forces on rings 2 to ring 9, $\:{F}_{\mathrm{r}}$, divided by the cross-sectional area of the specimen, $\:A$, for all the dense DEM specimens at 15% shear strain. The specimens with projection caps exhibited values of $\:\:{F}_{\mathrm{r}}/A$ close to zero, consistent with the small values expected in physical experiments. In contrast, FC-D showed relatively large $\:{F}_{\mathrm{r}}/A$, with the magnitudes increasing to around 5 kPa near the caps. For comparison, the peak shear stress measured at the bottom boundary at 15% strain is 28 kPa for this specimen. The large ring forces indicate that the rings also contributed to shear force transmission, which deviates the actual experimental mechanism and resulted in the lower shear stress magnitudes measured at the bottom boundary, as shown in Fig. 8a (Fig. 9).

## Particle displacements and rotations during shearing

**Fig. 10:**
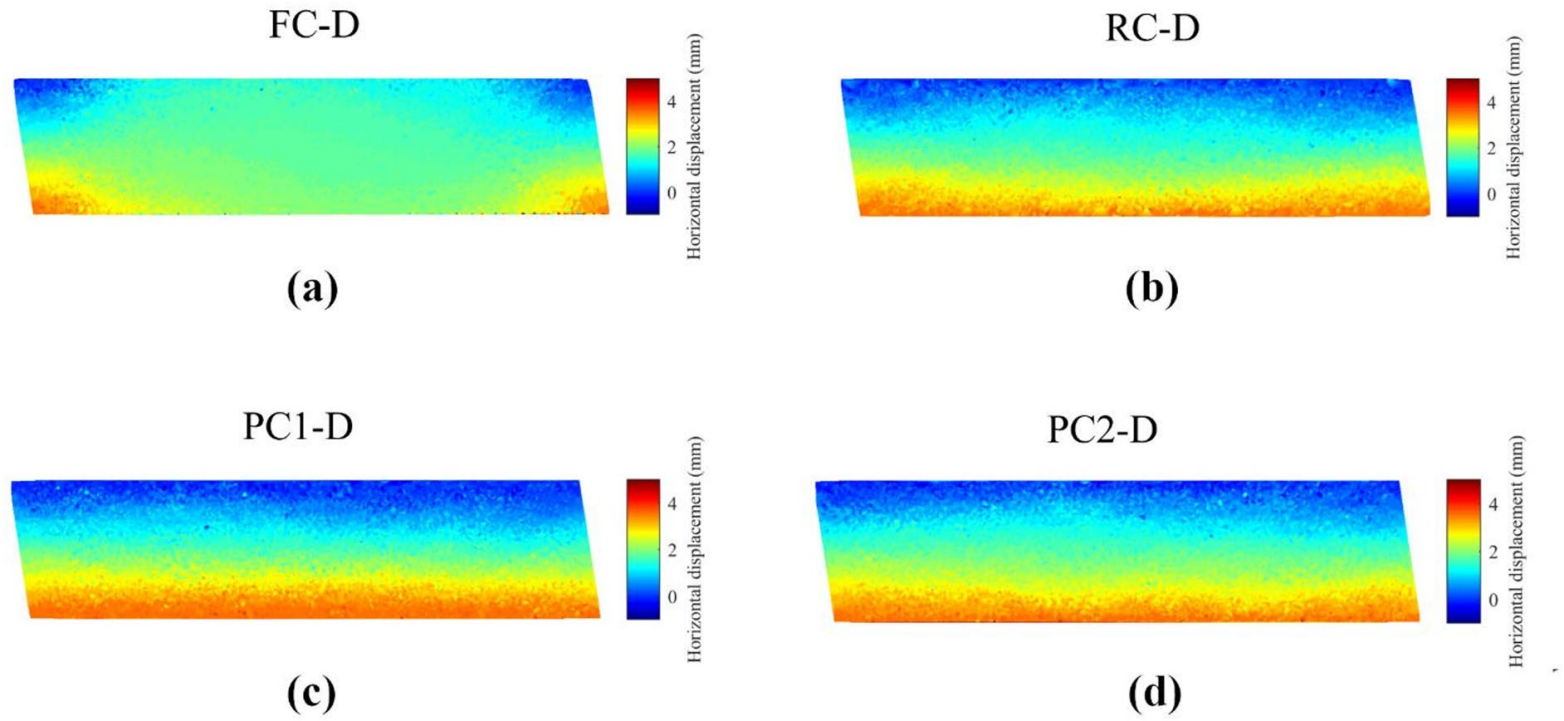
Cumulative horizontal displacement at 15% shear strain for dense specimens with different caps.

**Fig. 11:**
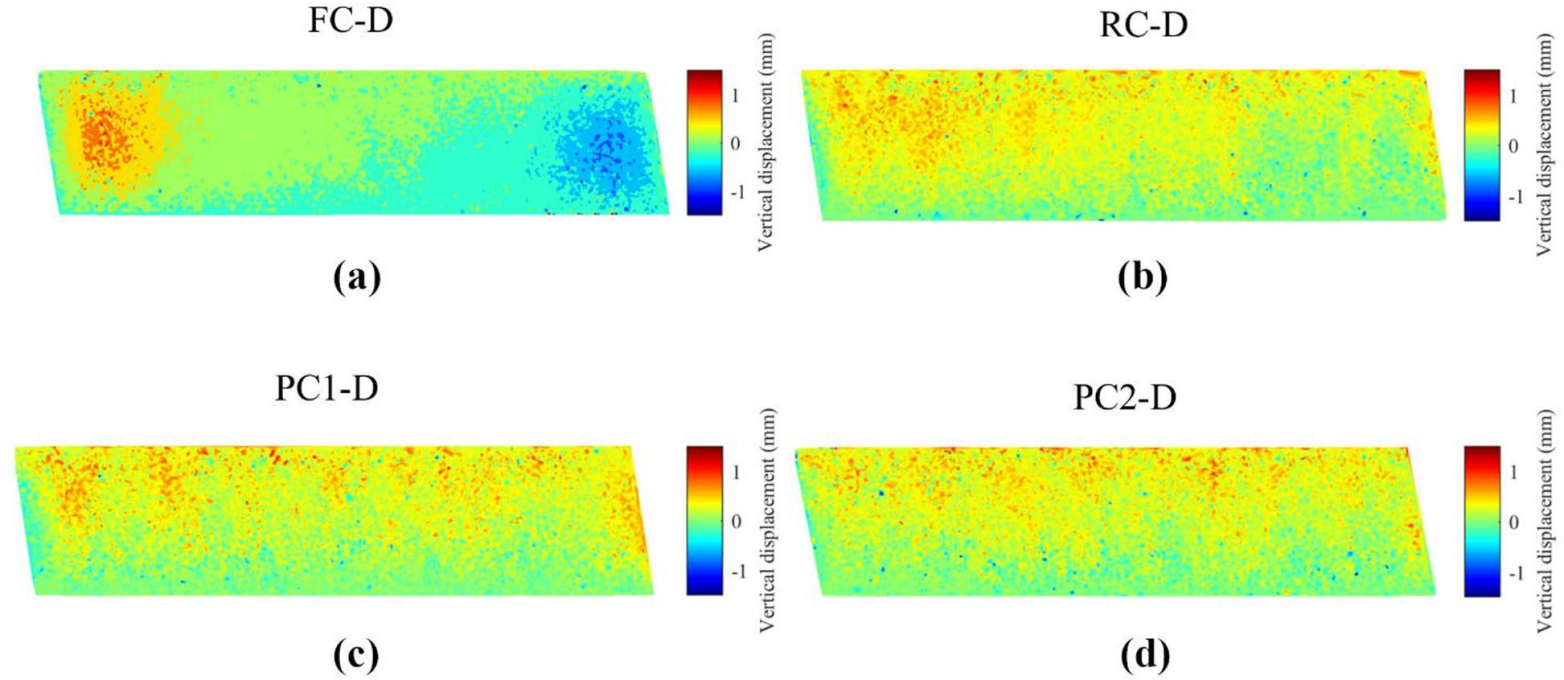
Cumulative vertical displacement at 15% shear strain for dense specimens with different caps.

The DEM simulations were analyzed from a microscopic point of view by studying particle movements and rotations. For a clear visualization of particle movements, a horizontal section of each specimen was taken from the middle, with a thickness of one-fifth of the diameter (20.32 mm), comprising more than 10,000 particles. Cumulative particle movements at 15% shear strain were obtained by comparing particle locations to their initial positions. Horizontal particle movements in dense specimens are depicted in Fig. 10, while vertical movements are shown in Fig. 11.

Figure 10 displays an elevation view of the specimens at $\:\gamma\:$ = 15%. Given a specimen height of around 25 mm, the maximum displacement at the bottom boundary at this point is approximately 3.75 mm. While all specimens had a linear profile due to the strain-controlled ring approach, distinct patterns of horizontal particle movements were observed, as shown in Fig. 10. The FC-D specimen notably differed from others, with large particle displacements observed only at the front and the back of the specimen, as defined along the shearing direction of the bottom cap. On the top cap of FC-D, particles with no horizontal movements were only found at edges close to the side rings, with uniform particle displacements elsewhere. This indicates that although the boundary displacement profile was forced to be linear, the particles inside the specimen did not exhibit a corresponding linear increase in horizontal displacement, which violates the fundamental mechanism of simple shearing. This phenomenon suggests that the flat cap did not efficiently transmit shear stress to the particles at the top and bottom boundaries, leading to slippage at the interfaces and preventing a linear horizontal displacement profile from forming within the specimen.

Specimens with projection boundaries achieved a simple shear condition, evidenced by incrementally increasing horizontal particle movements from top to bottom. Particles in contact with the bottom caps moved with the boundaries, with horizontal displacements of around 3.75 mm, consistent with the boundary displacement at $\:\gamma\:$ = 15%. Comparing PC1-D to RC-D and PC2-D, thicker layers of particles moving along with the boundaries were observed due to the larger projections, showing the effectiveness of projections in interlocking with particles, transmitting shear forces and ensuring simple shear conditions within specimens.

The overall contractive and dilative response of a specimen in simple shear experiments is reflected in the vertical movement of the top cap. The DEM simulations enable visualization of particle displacements in the vertical direction, influencing volumetric strain changes. For an idealized uniform specimen, achieving the volumetric strain shown in Fig. 8b would require vertical cap displacements of approximately 0.15 mm for FC-D, 0.23 mm for PC2-D, and 0.25 mm for PC1-D and RC-D. Figure 11 illustrates that the specimens RC-D, PC1-D and PC2-D displayed a similar pattern with relatively uniform vertical displacements at a certain height. In contrast, FC-D showed distinctive particle movements, with particles divided into two groups along an inclined line across the top and bottom of the specimen: particles moved downward at the front end in the direction of shearing and upward at the back end. Overall, the distribution of displacements resembles a combined active/passive wedge failure depending on whether a boundary was moving away from the specimen or into it, which is consistent with the rings acting as substantial contributors to the force transmission as confirmed by Fig. 9. This non-uniform distribution of vertical particle movements also suggests that the flat caps did not effectively transmit shear stress to the specimen. Although DEM simulations using flat caps indicated a microscopic response not characteristic of simple shear, the macroscopic behavior was relatively similar to the others. Therefore, solely examining experiments or macroscopic responses may not confirm the existence of simple shear conditions within the specimens. Unrealistic friction at the boundaries may result ambiguous DEM simulations.

**Fig. 12:**
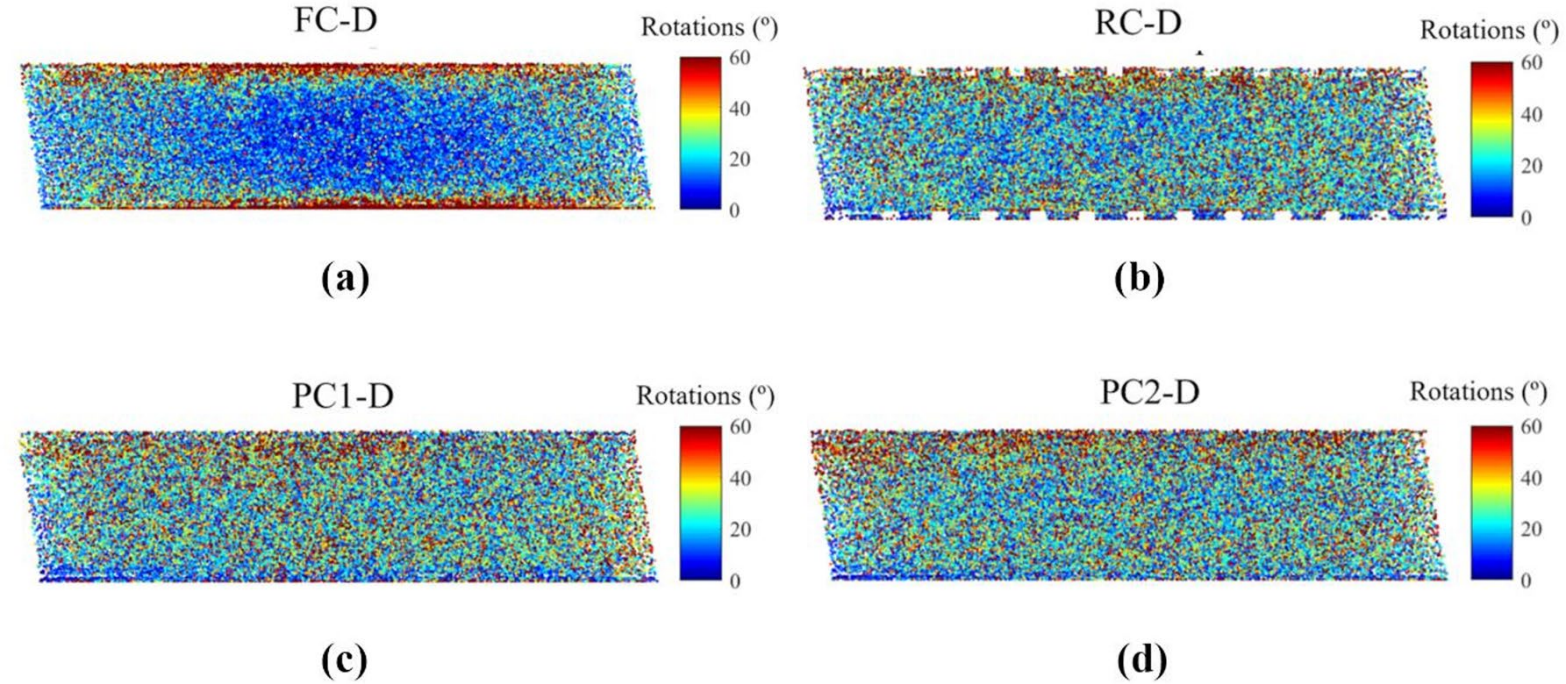
Absolute cumulative particle rotations.

Cumulative particle rotations were also calculated, as shown in Fig. 12. Large rotations are found along the boundaries of FC-D, confirming that the high-friction boundary caused particles to roll effectively amounting to slipping at the caps. Zero rolling friction is used to model spherical particles, which in turn allows easier rotation compared to sliding along the rough boundary. Therefore, particles more easily rotated when the relative movement of particles occurred. The distribution of relatively small particle rotations within the central portion of the specimen in Fig. 12a correlated with the particle horizontal movements in Fig. 10a, highlighting the violation of the simple shear condition. RC-D, PC1-D and PC2-D displayed similar patterns of particle rotations within the specimens. Distinct from FC-D, these specimens exhibited particle rotations across the entire specimen, indicating substantial particle engagement in shear displacement and force transmission.

## Conclusions

In the context of simple shear tests, the top and bottom boundaries play important roles in transmitting shear stress. For spherical particles such as steel bearings, flat boundaries, even with high-friction, lead to particle rolling and essentially boundary slippage, resulting in inadequate shear transmission capability. To address this challenge, three different boundary types- ribbed, large pyramid and small pyramid projections- were employed in experimental simple shear tests, demonstrating satisfactory performance in shear stress transmission. Similarly, DEM simulations were conducted using identical projection boundaries and experimental setups, showing good agreement with experimental outcomes. Additionally, DEM models applying flat caps with artificially enhanced boundary friction coefficients were also examined and compared against validated DEM simulations with projection boundaries. Key conclusions are as follows:

1. Caps with projections are effective in transmitting shear stresses and ensuring simple shear conditions through the specimens, as demonstrated by both physical tests and numerical simulations. DEM specimens with projection caps of various designs exhibited similar macroscopic responses, consistent with those observed in experiments, and showed similar microscopic responses in terms of particle displacements and rotations.
2. Although introducing projections increased the number of input meshes in DEM models, the increase in computational time was not as much as initially expected. Specifically, for the small pyramid projections employed in this study with the largest number of meshes, computational time increased by approximately 6%.
3. DEM simulations using flat boundaries with enhanced friction resulted in large particle rotations at the boundaries indicating poor shear stress transmission. The displacement pattern of the particles indicated an internal wedge-shaped failure, more consistent with active-passive modes than with simple shear. The analysis of microscopic response revealed that it is unsuitable to change the interparticle friction to match the macroscopic experimental results, as it can provide misleading responses of specimens.
4. The macroscopic response of specimens using flat boundaries with enhanced friction showed lower values of peak shear stress and initial stiffness compared to simulations using boundaries with projections, but overall did not provide a clear indication of the unrealistic internal response. Calibrating the boundary friction of DEM simulations with experimental results at the macroscopic level may lead to substantial errors in capturing the correct failure mechanism, even if the overall values appear to be consistent.
5. Since only boundary measurements are possible for physical specimens, the actual void ratio in the portion of the specimen that is responding in simple shear cannot be assessed. However, though experimental calculation of void ratio may be subject to significant errors, comparison of physical and numerical experiments showed that the range of expected responses in the dense-to-loose range remain consistent.

While this study focused on steel bearing specimens, its implications are extendable to other granular materials and boundary-driven problems. The recommendation is to incorporate projection-based top and bottom caps in both experimental and numerical simple shear tests to ensure effective shear transmission. The 3D printing technology used in this study enables the manufacturing of caps with various features. For experiments with different manufacturing technologies, ribbed features are easier to machine compared to the pyramid projections. However, the pyramid features are preferable in multi-directional tests attributed to their uniform response under different loading directions.

## Data Availability

All data generated or analysed during this study are included in this article.

## List of symbols

$$\:\rho\:$$: Particle density
$\:E$: Young’s modulus of steel particles
$\:{\upnu\:}$: Poisson’s ratio of steel particles
$\:{\mu\:}_{p}$: Interparticle friction coefficient
$\:{\mu\:}_{\mathrm{r}}$: Coefficient of friction for particle-ring interface
$\:{\mu\:}_{\mathrm{c}}$: Coefficient of friction for particle-cap interface
$$\:t$$: Timestep
$\:\dot{{\varepsilon}_{q}}$: Shear strain rate
$\:{e}_{0}$: Initial void ratio
$\:{e}_{\mathrm{i}}$: Void ratio of each layer within a specimen
$$\:\tau\:$$: Shear stress
$$\:{\varepsilon}_{v}$$: Volumetric strain
$$\:\gamma\:$$: Shear strain
$\:{F}_{\mathrm{r}}$: Forces on confining rings
$\:A$: Cross-sectional area of the specimen
